# 2-Amino-3-carb­oxy­pyridinium chloride hemihydrate

**DOI:** 10.1107/S1600536812017230

**Published:** 2012-04-21

**Authors:** Rafika Bouchene, Sofiane Bouacida, Fadila Berrah, Jean-Claude Daran

**Affiliations:** a Laboratoire de Chimie Appliquée et Technologie des Matériaux (LCATM), Université Oum El Bouaghi, Algeria; b Département Sciences de la Matière, Faculté des Sciences Exactes et Sciences de la Nature et de la Vie, Université Oum El Bouaghi, Algeria; c Unité de Recherche de Chimie de l’Environnement et Moléculaire Structurale (CHEMS), Université Mentouri–Constantine, 25000 Algeria; d Laboratoire de Chimie de Coodination, UPR–CNRS 8241, 205 route de Narbonne, 31077 Toulouse Cedex 04, France

## Abstract

The asymmetric unit of the title compound, C_6_H_7_N_2_O_2_^+^·Cl^−^·0.5H_2_O, consists of two protonated 2-amino-3-carb­oxy­pyridine cations, two chloride anions and one mol­ecule of water. The crystal packing can be described as alternating layers of cations and anions parallel to (110), which are linked together by O_w_—H⋯Cl inter­actions. In the crystal, four types of classical hydrogen bonds are observed, *viz*. cation–anion (O—H⋯Cl and N—H⋯Cl), cation–cation (N—H⋯O), cation–water (N—H⋯O_w_) and water–anion (O_w_—H⋯Cl), resulting in the formation of an infinite three-dimensional network.

## Related literature

For applications of hybrid organic–inorganic compounds, see: Bouacida (2008 ▶); Kickelbick (2007 ▶); Mitzi *et al.* (1998 ▶); Asaji *et al.* (2007 ▶); Lynch & Jones (2004 ▶). For related structures, see: Beatty (2003 ▶); Sengupta *et al.* (2001 ▶); Berrah *et al.* (2011*a* ▶,*b* ▶,*c* ▶); Akriche & Rzaigui (2007 ▶). 
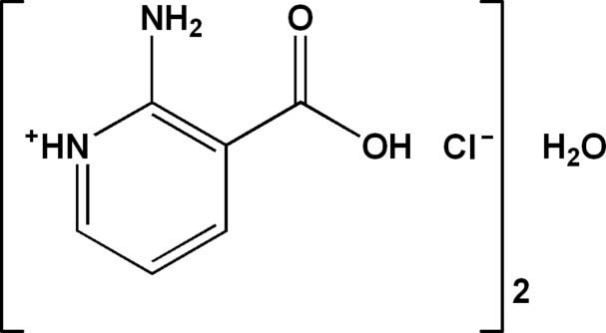


## Experimental

### 

#### Crystal data

C_6_H_7_N_2_O_2_^+^·Cl^−^·0.5H_2_O
*M* *_r_* = 183.60
Triclinic, 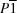

*a* = 7.8949 (4) Å
*b* = 9.1639 (5) Å
*c* = 11.0285 (6) Å
α = 81.392 (4)°
β = 81.276 (3)°
γ = 81.682 (4)°
*V* = 773.68 (7) Å^3^
*Z* = 4
Mo *K*α radiation
μ = 0.45 mm^−1^
*T* = 180 K
0.1 × 0.08 × 0.06 mm

#### Data collection

Agilent Xcalibur Sapphire1 long-nozzle diffractometer
Absorption correction: multi-scan (*CrysAlis PRO*; Agilent, 2011 ▶) *T*_min_ = 0.831, *T*_max_ = 1
14449 measured reflections
3600 independent reflections
2857 reflections with *I* > 2σ(*I*)
*R*_int_ = 0.033

#### Refinement

*R*[*F* ^2^ > 2σ(*F* ^2^)] = 0.030
*wR*(*F* ^2^) = 0.08
*S* = 1.01
3600 reflections
214 parameters
3 restraints
H atoms treated by a mixture of independent and constrained refinement
Δρ_max_ = 0.26 e Å^−3^
Δρ_min_ = −0.28 e Å^−3^

### 

Data collection: *CrysAlis PRO* (Agilent, 2011 ▶); cell refinement: *CrysAlis PRO*; data reduction: *CrysAlis PRO*; program(s) used to solve structure: *SIR2002* (Burla *et al.*, 2005 ▶); program(s) used to refine structure: *SHELXL97* (Sheldrick, 2008 ▶); molecular graphics: *ORTEP-3 for Windows* (Farrugia,1997 ▶) and *DIAMOND* (Brandenburg & Berndt, 2001 ▶); software used to prepare material for publication: *WinGX* (Farrugia, 1999 ▶).

## Supplementary Material

Crystal structure: contains datablock(s) global, I. DOI: 10.1107/S1600536812017230/bq2352sup1.cif

Structure factors: contains datablock(s) I. DOI: 10.1107/S1600536812017230/bq2352Isup2.hkl

Supplementary material file. DOI: 10.1107/S1600536812017230/bq2352Isup3.cml

Additional supplementary materials:  crystallographic information; 3D view; checkCIF report

## Figures and Tables

**Table 1:** Hydrogen-bond geometry (Å, °)

*D*—H⋯*A*	*D*—H	H⋯*A*	*D*⋯*A*	*D*—H⋯*A*
O1*W*—H1*W*⋯Cl1	0.85 (1)	2.24 (1)	3.0887 (12)	173 (2)
O1*W*—H2*W*⋯Cl2	0.84 (2)	2.33 (2)	3.1639 (12)	170 (2)
O2*A*—H2*A*⋯Cl1^i^	0.82	2.18	2.9948 (11)	177
O2*B*—H2*B*⋯O1*W*^i^	0.82	1.78	2.5818 (15)	166
N3*B*—H3*B*2⋯O1*B*	0.86	2.10	2.7176 (17)	128
N3*B*—H3*B*2⋯O1*A*^ii^	0.86	2.25	2.9903 (17)	144
N3*A*—H3*A*2⋯O1*A*	0.86	2.04	2.6644 (16)	129
N3*A*—H3*A*2⋯O1*B*^ii^	0.86	2.17	2.8781 (17)	140
N3*A*—H3*A*1⋯Cl2^iii^	0.86	2.34	3.1447 (13)	156
N4*A*—H4*A*⋯Cl2^iii^	0.86	2.44	3.2265 (12)	152
N4*B*—H4*B*⋯Cl2	0.86	2.21	3.0510 (13)	166

Symmetry codes: (i) 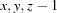; (ii) 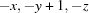; (iii) 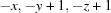.

**Fig. 1.**
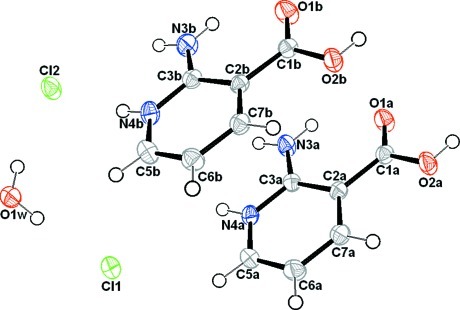
The asymmetric unit of (I) with the atom-labeling scheme. Displacement ellipsoids are drawn at the 50% probability level.

**Fig. 2.**
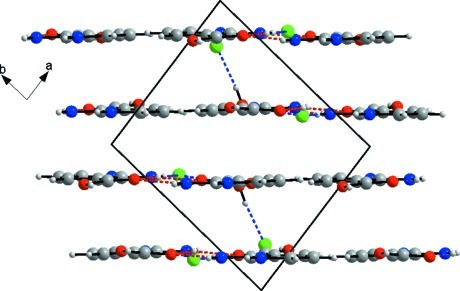
(Brandenburg & Berndt, 2001) Partial packing viewed via c axis showing layers parallel to (110) plane, which are connected with O—H···Cl Hydrogen bonds, shown as dashed lines.

**Fig. 3.**
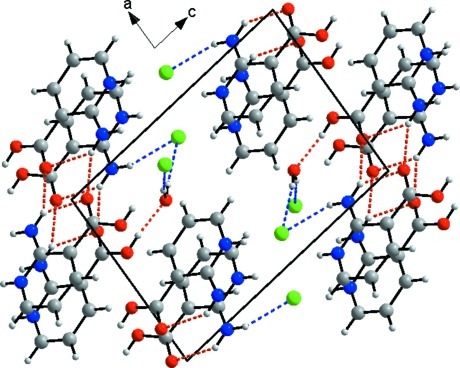
(Brandenburg & Berndt, 2001) Partial packing viewed via b axis showing Hydrogen bonds interactions, as dashed lines, cation-anion [O—H···Cl & N—H···Cl], cation-cation [N—H···O], cation-water [N—H···O1W] and water-anion [O1W—H···Cl].

## supplementary crystallographic information

### Comment

Organic-inorganic hybrid compounds represent one of the most important developments in materials chemistry in recent years. The tremendous possibilities of combination of different properties in one material initiated an explosion of ideas about potential materials and applications (Bouacida, 2008; Kickelbick, 2007; Mitzi *et al.*, 1998). Hybrid structures including substituted pyridines organic units have drawn increasing attention due to their potential applications in biological and industrial fields (Asaji *et al.*, 2007; Lynch & Jones, 2004), nitrogen in the pyridine ring has a lone pair of electrons which is not delocalized with the aromatic π-electron system and is easily available for protonation (Berrah *et al.*, 2011*a*). In the presence of a carboxylic acid substituent, they are recognized as efficient N–O donors exhibiting diverse mode of coordination (Beatty, 2003; Sengupta *et al.*, 2001). Their fascinating structures are rich in H-bonds wich have a potential importance in crystal stability (Berrah *et al.*, 2011*a*,*b*,*c*; Akriche & Rzaigui, 2007).

In continuation of our search to enrich the varieties in such kinds of hybrid compounds and to investigate the influence of hydrogen bonds on the structural features, we report here the synthesis and crystal structure of 2-amino-3-carboxypyridinium chloride hemi hydrate, (I).

The asymmetric unit in this compound consists of two protonated, "2-amino-3-carboxypyridine", amino acids cations (A and B), two chloride anions and one molecule of water. The molecular geometry and the atom-numbering scheme of (I) are shown in Fig. 1. Bond distances and angles observed in the different entities, present no unusual features and are consistent with those reported previously (Berrah *et al.*, 2011*b*). The crystal packing can be described as alternating layers parallel to (110) plane, which are linked together by O1W—H···Cl interactions involving molecule of water and anions chloride (Fig.2). In this structure, four types of classical hydrogen bonds are observed, *viz*. cation-anion [O—H···Cl & N—H···Cl], cation-cation [N—H···O], cation-water [N—H···O1W] and water-anion [O1W—H···Cl] (Fig. 3). All these interactions bonds link the molecules within the layers and also link the layers together, forming a three-dimensional network and reinforcing the cohesion of the structure. Additional hydrogen bond parameters are listed in table 1.

### Experimental

The title compound was synthesized by reacting 3-amino-pyridine-2-carboxylic acid (3 mmol) with InCl_3_ (1 mmol)in an aqueous solution of hydrochloric acid. The solutions were slowly evaporated to dryness for a couple of weeks. Some colorless crystals were carefully isolated under polarizing microscope for analysis by X-ray diffraction.

### Refinement

The H atoms were localized on Fourier maps but introduced in calculated positions and treated as riding on their parent atoms (C, N or O) with C—H = 0.93 Å, O—H = 0.82 Å and N—H = 0.86 Å with *U*_iso_(H) = 1.2 *U*_eq_(C or N) and *U*_iso_(H) = 1.5 *U*_eq_(O). H1W and H2W were located in a difference Fourier map and refined isotropically with *U*_iso_(H) = 1.5*U*_eq_(O).

### Crystal data

**Table:**

C_6_H_7_N_2_O_2_^+^·Cl^−^·0.5H_2_O	*Z* = 4
*M**_r_* = 183.60	*F*(000) = 380
Triclinic, *P*1	*D*_x_ = 1.576 Mg m^−^^3^
*a* = 7.8949 (4) Å	Mo *K*α radiation, λ = 0.71073 Å
*b* = 9.1639 (5) Å	Cell parameters from 8921 reflections
*c* = 11.0285 (6) Å	θ = 3.0–28.3°
α = 81.392 (4)°	µ = 0.45 mm^−^^1^
β = 81.276 (3)°	*T* = 180 K
γ = 81.682 (4)°	Box, colourless
*V* = 773.68 (7) Å^3^	0.1 × 0.08 × 0.06 mm

### Data collection

**Table:**

Agilent Xcalibur Sapphire1 long-nozzle diffractometer	3600 independent reflections
Radiation source: fine-focus sealed tube	2857 reflections with *I* > 2σ(*I*)
Graphite monochromator	*R*_int_ = 0.033
Detector resolution: 8.2632 pixels mm^-1^	θ_max_ = 28.4°, θ_min_ = 3.0°
ω scans	*h* = −10→9
Absorption correction: multi-scan (*CrysAlis PRO*; Agilent, 2011)	*k* = −12→12
*T*_min_ = 0.831, *T*_max_ = 1	*l* = −13→14
14449 measured reflections	

### Refinement

**Table:**

Refinement on *F*^2^	Primary atom site location: structure-invariant direct methods
Least-squares matrix: full	Secondary atom site location: difference Fourier map
*R*[*F*^2^ > 2σ(*F*^2^)] = 0.030	Hydrogen site location: inferred from neighbouring sites
*wR*(*F*^2^) = 0.08	H atoms treated by a mixture of independent and constrained refinement
*S* = 1.01	*w* = 1/[σ^2^(*F*_o_^2^) + (0.0442*P*)^2^ + 0.1332*P*] where *P* = (*F*_o_^2^ + 2*F*_c_^2^)/3
3600 reflections	(Δ/σ)_max_ = 0.006
214 parameters	Δρ_max_ = 0.26 e Å^−^^3^
3 restraints	Δρ_min_ = −0.28 e Å^−^^3^

### Special details

**Table:**

Geometry. All e.s.d.'s (except the e.s.d. in the dihedral angle between two l.s. planes) are estimated using the full covariance matrix. The cell e.s.d.'s are taken into account individually in the estimation of e.s.d.'s in distances, angles and torsion angles; correlations between e.s.d.'s in cell parameters are only used when they are defined by crystal symmetry. An approximate (isotropic) treatment of cell e.s.d.'s is used for estimating e.s.d.'s involving l.s. planes.
Refinement. Refinement of *F*^2^ against ALL reflections. The weighted *R*-factor *wR* and goodness of fit *S* are based on *F*^2^, conventional *R*-factors *R* are based on *F*, with *F* set to zero for negative *F*^2^. The threshold expression of *F*^2^ > σ(*F*^2^) is used only for calculating *R*-factors(gt) *etc*. and is not relevant to the choice of reflections for refinement. *R*-factors based on *F*^2^ are statistically about twice as large as those based on *F*, and *R*- factors based on ALL data will be even larger.

### Fractional atomic coordinates and isotropic or equivalent isotropic displacement parameters (Å^2^)

**Table:**

	*x*	*y*	*z*	*U*_iso_*/*U*_eq_	
N3A	−0.02482 (16)	0.24894 (14)	0.18532 (11)	0.0277 (3)	
H3A1	−0.0772	0.2962	0.2448	0.033*	
H3A2	−0.0497	0.2761	0.1114	0.033*	
N3B	0.13489 (17)	0.59931 (14)	0.23112 (12)	0.0291 (3)	
H3B1	0.0857	0.6432	0.2933	0.035*	
H3B2	0.1053	0.6298	0.1588	0.035*	
O1W	0.33167 (15)	0.38876 (12)	0.72134 (10)	0.0306 (2)	
H1W	0.287 (2)	0.3146 (14)	0.7070 (17)	0.046*	
H2W	0.280 (2)	0.4650 (14)	0.6834 (16)	0.046*	
Cl1	0.19706 (5)	0.11808 (4)	0.64870 (3)	0.03012 (11)	
Cl2	0.14081 (5)	0.65043 (4)	0.55111 (3)	0.03071 (11)	
O1A	0.05136 (14)	0.18895 (12)	−0.04721 (9)	0.0301 (2)	
O2A	0.26652 (14)	0.01182 (12)	−0.09112 (9)	0.0287 (2)	
H2A	0.2445	0.0392	−0.1619	0.043*	
O1B	0.20803 (14)	0.54951 (12)	−0.00916 (10)	0.0333 (3)	
N4A	0.12830 (15)	0.09603 (13)	0.32534 (10)	0.0220 (2)	
H4A	0.074	0.1486	0.3809	0.026*	
N4B	0.29795 (16)	0.44117 (14)	0.36162 (11)	0.0259 (3)	
H4B	0.2425	0.488	0.4204	0.031*	
C2B	0.34601 (17)	0.40270 (14)	0.15203 (12)	0.0193 (3)	
C2A	0.19059 (17)	0.05053 (15)	0.11658 (12)	0.0196 (3)	
C3A	0.09315 (17)	0.13510 (15)	0.20751 (12)	0.0201 (3)	
C5A	0.24283 (19)	−0.01981 (16)	0.36061 (13)	0.0256 (3)	
H5A	0.2593	−0.0421	0.4434	0.031*	
C7A	0.30817 (19)	−0.06680 (16)	0.15319 (13)	0.0238 (3)	
H7A	0.372	−0.1227	0.0942	0.029*	
C1A	0.16107 (18)	0.09171 (15)	−0.01399 (12)	0.0210 (3)	
O2B	0.38993 (13)	0.34859 (11)	−0.05118 (9)	0.0272 (2)	
H2B	0.3638	0.3747	−0.1211	0.041*	
C1B	0.30608 (17)	0.44226 (15)	0.02359 (12)	0.0209 (3)	
C7B	0.47068 (18)	0.28766 (16)	0.18251 (13)	0.0227 (3)	
H7B	0.5304	0.2339	0.1208	0.027*	
C3B	0.25557 (18)	0.48538 (15)	0.24663 (13)	0.0218 (3)	
C5B	0.4206 (2)	0.32921 (17)	0.39066 (14)	0.0279 (3)	
H5B	0.4441	0.3064	0.4719	0.033*	
C6B	0.51055 (19)	0.24892 (17)	0.30269 (13)	0.0264 (3)	
H6B	0.5956	0.171	0.322	0.032*	
C6A	0.3345 (2)	−0.10448 (17)	0.27672 (14)	0.0281 (3)	
H6A	0.413	−0.1856	0.3008	0.034*	

### Atomic displacement parameters (Å^2^)

**Table:**

	*U*^11^	*U*^22^	*U*^33^	*U*^12^	*U*^13^	*U*^23^
N3A	0.0289 (7)	0.0298 (7)	0.0217 (6)	0.0094 (6)	−0.0026 (5)	−0.0084 (5)
N3B	0.0302 (7)	0.0268 (6)	0.0280 (7)	0.0051 (6)	−0.0008 (5)	−0.0080 (5)
O1W	0.0410 (7)	0.0254 (6)	0.0259 (5)	0.0005 (5)	−0.0104 (5)	−0.0036 (4)
Cl1	0.0377 (2)	0.0322 (2)	0.02155 (18)	−0.00542 (16)	−0.00607 (15)	−0.00379 (14)
Cl2	0.0382 (2)	0.0302 (2)	0.02172 (18)	0.00677 (16)	−0.00392 (15)	−0.00771 (14)
O1A	0.0310 (6)	0.0335 (6)	0.0227 (5)	0.0100 (5)	−0.0058 (4)	−0.0049 (4)
O2A	0.0349 (6)	0.0301 (6)	0.0179 (5)	0.0084 (5)	−0.0028 (4)	−0.0059 (4)
O1B	0.0351 (6)	0.0326 (6)	0.0278 (6)	0.0113 (5)	−0.0065 (5)	−0.0024 (4)
N4A	0.0249 (6)	0.0228 (6)	0.0185 (5)	−0.0021 (5)	−0.0001 (5)	−0.0062 (4)
N4B	0.0261 (6)	0.0306 (7)	0.0208 (6)	−0.0008 (5)	0.0008 (5)	−0.0093 (5)
C2B	0.0190 (6)	0.0178 (6)	0.0209 (6)	−0.0034 (5)	−0.0004 (5)	−0.0032 (5)
C2A	0.0190 (7)	0.0188 (6)	0.0209 (7)	−0.0021 (5)	−0.0015 (5)	−0.0042 (5)
C3A	0.0193 (7)	0.0216 (7)	0.0197 (6)	−0.0041 (5)	−0.0017 (5)	−0.0033 (5)
C5A	0.0304 (8)	0.0265 (7)	0.0202 (7)	−0.0028 (6)	−0.0061 (6)	−0.0014 (6)
C7A	0.0250 (7)	0.0223 (7)	0.0232 (7)	0.0005 (6)	−0.0013 (6)	−0.0055 (5)
C1A	0.0211 (7)	0.0212 (7)	0.0204 (7)	−0.0028 (6)	−0.0003 (5)	−0.0045 (5)
O2B	0.0330 (6)	0.0277 (5)	0.0197 (5)	0.0050 (5)	−0.0058 (4)	−0.0058 (4)
C1B	0.0191 (7)	0.0204 (7)	0.0225 (7)	−0.0017 (6)	−0.0007 (5)	−0.0039 (5)
C7B	0.0224 (7)	0.0227 (7)	0.0232 (7)	−0.0025 (6)	−0.0009 (6)	−0.0058 (5)
C3B	0.0201 (7)	0.0217 (7)	0.0239 (7)	−0.0049 (6)	−0.0005 (5)	−0.0043 (5)
C5B	0.0284 (8)	0.0344 (8)	0.0210 (7)	−0.0043 (7)	−0.0050 (6)	−0.0024 (6)
C6B	0.0247 (7)	0.0269 (7)	0.0264 (7)	0.0007 (6)	−0.0059 (6)	−0.0013 (6)
C6A	0.0300 (8)	0.0249 (7)	0.0273 (7)	0.0049 (6)	−0.0070 (6)	−0.0018 (6)

### Geometric parameters (Å, º)

**Table:**

N3A—C3A	1.3143 (18)	C2B—C7B	1.3738 (19)
N3A—H3A1	0.86	C2B—C3B	1.4236 (19)
N3A—H3A2	0.86	C2B—C1B	1.4785 (18)
N3B—C3B	1.3172 (18)	C2A—C7A	1.3695 (19)
N3B—H3B1	0.86	C2A—C3A	1.4227 (19)
N3B—H3B2	0.86	C2A—C1A	1.4778 (18)
O1W—H1W	0.854 (9)	C5A—C6A	1.353 (2)
O1W—H2W	0.843 (9)	C5A—H5A	0.93
O1A—C1A	1.2058 (17)	C7A—C6A	1.394 (2)
O2A—C1A	1.3221 (16)	C7A—H7A	0.93
O2A—H2A	0.82	O2B—C1B	1.3179 (16)
O1B—C1B	1.2058 (17)	O2B—H2B	0.82
N4A—C5A	1.3420 (18)	C7B—C6B	1.3911 (19)
N4A—C3A	1.3548 (17)	C7B—H7B	0.93
N4A—H4A	0.86	C5B—C6B	1.357 (2)
N4B—C5B	1.342 (2)	C5B—H5B	0.93
N4B—C3B	1.3491 (18)	C6B—H6B	0.93
N4B—H4B	0.86	C6A—H6A	0.93
			
C3A—N3A—H3A1	120	C2A—C7A—C6A	121.60 (13)
C3A—N3A—H3A2	120	C2A—C7A—H7A	119.2
H3A1—N3A—H3A2	120	C6A—C7A—H7A	119.2
C3B—N3B—H3B1	120	O1A—C1A—O2A	123.10 (12)
C3B—N3B—H3B2	120	O1A—C1A—C2A	123.28 (12)
H3B1—N3B—H3B2	120	O2A—C1A—C2A	113.62 (12)
H1W—O1W—H2W	106.3 (15)	C1B—O2B—H2B	109.5
C1A—O2A—H2A	109.5	O1B—C1B—O2B	123.72 (13)
C5A—N4A—C3A	123.91 (12)	O1B—C1B—C2B	123.43 (12)
C5A—N4A—H4A	118	O2B—C1B—C2B	112.84 (12)
C3A—N4A—H4A	118	C2B—C7B—C6B	122.19 (13)
C5B—N4B—C3B	124.58 (13)	C2B—C7B—H7B	118.9
C5B—N4B—H4B	117.7	C6B—C7B—H7B	118.9
C3B—N4B—H4B	117.7	N3B—C3B—N4B	118.05 (13)
C7B—C2B—C3B	118.77 (12)	N3B—C3B—C2B	125.61 (13)
C7B—C2B—C1B	121.29 (12)	N4B—C3B—C2B	116.33 (13)
C3B—C2B—C1B	119.94 (12)	N4B—C5B—C6B	120.70 (13)
C7A—C2A—C3A	118.82 (12)	N4B—C5B—H5B	119.7
C7A—C2A—C1A	122.21 (12)	C6B—C5B—H5B	119.7
C3A—C2A—C1A	118.96 (12)	C5B—C6B—C7B	117.41 (14)
N3A—C3A—N4A	118.07 (12)	C5B—C6B—H6B	121.3
N3A—C3A—C2A	125.06 (12)	C7B—C6B—H6B	121.3
N4A—C3A—C2A	116.85 (12)	C5A—C6A—C7A	118.20 (14)
N4A—C5A—C6A	120.56 (13)	C5A—C6A—H6A	120.9
N4A—C5A—H5A	119.7	C7A—C6A—H6A	120.9
C6A—C5A—H5A	119.7		
			
C5A—N4A—C3A—N3A	178.44 (13)	C7B—C2B—C1B—O2B	5.75 (18)
C5A—N4A—C3A—C2A	−2.8 (2)	C3B—C2B—C1B—O2B	−174.82 (12)
C7A—C2A—C3A—N3A	−179.18 (14)	C3B—C2B—C7B—C6B	0.4 (2)
C1A—C2A—C3A—N3A	0.5 (2)	C1B—C2B—C7B—C6B	179.82 (13)
C7A—C2A—C3A—N4A	2.20 (19)	C5B—N4B—C3B—N3B	−178.55 (13)
C1A—C2A—C3A—N4A	−178.08 (12)	C5B—N4B—C3B—C2B	1.8 (2)
C3A—N4A—C5A—C6A	1.4 (2)	C7B—C2B—C3B—N3B	179.06 (13)
C3A—C2A—C7A—C6A	−0.2 (2)	C1B—C2B—C3B—N3B	−0.4 (2)
C1A—C2A—C7A—C6A	−179.93 (14)	C7B—C2B—C3B—N4B	−1.36 (19)
C7A—C2A—C1A—O1A	175.54 (13)	C1B—C2B—C3B—N4B	179.19 (12)
C3A—C2A—C1A—O1A	−4.2 (2)	C3B—N4B—C5B—C6B	−1.3 (2)
C7A—C2A—C1A—O2A	−4.50 (19)	N4B—C5B—C6B—C7B	0.1 (2)
C3A—C2A—C1A—O2A	175.79 (12)	C2B—C7B—C6B—C5B	0.3 (2)
C7B—C2B—C1B—O1B	−173.77 (14)	N4A—C5A—C6A—C7A	0.8 (2)
C3B—C2B—C1B—O1B	5.7 (2)	C2A—C7A—C6A—C5A	−1.3 (2)

### Hydrogen-bond geometry (Å, º)

**Table:**

*D*—H···*A*	*D*—H	H···*A*	*D*···*A*	*D*—H···*A*
O1*W*—H1*W*···Cl1	0.85 (1)	2.24 (1)	3.0887 (12)	173 (2)
O1*W*—H2*W*···Cl2	0.84 (2)	2.33 (2)	3.1639 (12)	170 (2)
O2*A*—H2*A*···Cl1^i^	0.82	2.18	2.9948 (11)	177
O2*B*—H2*B*···O1*W*^i^	0.82	1.78	2.5818 (15)	166
N3*B*—H3*B*2···O1*B*	0.86	2.10	2.7176 (17)	128
N3*B*—H3*B*2···O1*A*^ii^	0.86	2.25	2.9903 (17)	144
N3*A*—H3*A*2···O1*A*	0.86	2.04	2.6644 (16)	129
N3*A*—H3*A*2···O1*B*^ii^	0.86	2.17	2.8781 (17)	140
N3*A*—H3*A*1···Cl2^iii^	0.86	2.34	3.1447 (13)	156
N4*A*—H4*A*···Cl2^iii^	0.86	2.44	3.2265 (12)	152
N4*B*—H4*B*···Cl2	0.86	2.21	3.0510 (13)	166
C5*A*—H5*A*···Cl1	0.93	2.82	3.5417 (15)	135
C6*A*—H6*A*···O1*W*^iv^	0.93	2.55	3.427 (2)	158
C7*A*—H7*A*···O2*A*	0.93	2.42	2.7397 (17)	100
C7*B*—H7*B*···O2*B*	0.93	2.37	2.7065 (17)	101

Symmetry codes: (i) *x*, *y*, *z*−1; (ii) −*x*, −*y*+1, −*z*; (iii) −*x*, −*y*+1, −*z*+1; (iv) −*x*+1, −*y*, −*z*+1.
